# The *SsDREB* Transcription Factor from the Succulent Halophyte *Suaeda salsa* Enhances Abiotic Stress Tolerance in Transgenic Tobacco

**DOI:** 10.1155/2015/875497

**Published:** 2015-10-04

**Authors:** Xu Zhang, Xiaoxue Liu, Lei Wu, Guihong Yu, Xiue Wang, Hongxiang Ma

**Affiliations:** ^1^Provincial Key Laboratory of Agrobiology, Institute of Biotechnology, Jiangsu Academy of Agricultural Sciences, Nanjing 210014, China; ^2^National Key Laboratory of Crop Genetics and Germplasm Enhancement, Cytogenetics Institute, Nanjing Agricultural University, Nanjing 210095, China

## Abstract

Dehydration-responsive element-binding (DREB) transcription factor (TF) plays a key role for abiotic stress tolerance in plants. In this study, a novel cDNA encoding DREB transcription factor, designated *SsDREB*, was isolated from succulent halophyte *Suaeda salsa*. This protein was classified in the A-6 group of DREB subfamily based on multiple sequence alignments and phylogenetic characterization. Yeast one-hybrid assays showed that SsDREB protein specifically binds to the DRE sequence and could activate the expression of reporter genes in yeast, suggesting that the SsDREB protein was a CBF/DREB transcription factor. Real-time RT-PCR showed that *SsDREB* was significantly induced under salinity and drought stress. Overexpression of *SsDREB* cDNA in transgenic tobacco plants exhibited an improved salt and drought stress tolerance in comparison to the nontransformed controls. The transgenic plants revealed better growth, higher chlorophyll content, and net photosynthesis rate, as well as higher level of proline and soluble sugars. The semiquantitative PCR of transgenics showed higher expression of stress-responsive genes. These data suggest that the *SsDREB* transcription factor is involved in the regulation of salt stress tolerance in tobacco by the activation of different downstream gene expression.

## 1. Introduction

The abiotic stresses like salinity, drought, and low and high temperature negatively affect plant growth and productivity [1]. They are major limiting factors for sustainable food production as they reduce yields by more than 50% in crop plants [2]. To overcome these limitations, plants have generated mechanisms to trigger a cascade of events leading to changes in gene expression and subsequently to biochemical physiological modifications that can enhance their stress tolerance [3]. Molecular and cellular responses to abiotic stresses involve signal perception, transduction of the signal to the cytoplasm and nucleus, alteration of gene expression and, finally, metabolic changes that lead to stress tolerance [4]. Numerous abiotic stress-related genes and transcription factors (TFs) have been isolated from different plant species and overexpressed in homologous and heterologous systems to engineer stress tolerance [5]. The Dehydration-responsive element-binding proteins (DREBs) are members of the APETALA2/ethylene-responsive element-binding factor (AP2/ERF) family of transcription factors in the promoters of stress-inducible genes [6].

Genes included in the DREB subfamily are divided into six small subgroups (A-1 to A-6) based on similarities in the binding domain. The A-1 subgroup, which includes the DREB1/CBF- (C-repeat binding factor-) like genes, are mainly induced by low temperature and activate the expression of many cold stress-responsive genes, whereas the A-2 subgroup, which is comprised of the DREB2 genes, mainly functions in osmotic stress [7]. In addition, multiple research reports indicated that the genes on the CBF/DREB family play very important roles in regulating abiotic stress via ABA-independent/dependent pathway [8–10]. It suggested that CBF/DREB plays distinctive roles in plant response to stress [11] and that there might also be a crosstalk between drought and cold responsive genes with a DRE element [6]. DREB2 homologous genes have been isolated from a variety of species [12]. Transgenic plants overexpressing either DREB1 or DREB2A genes enhanced tolerance to abiotic stress [13–16].

To date, only few efforts are made in halophytes in response to salt stress. The expression of AhDREB1 from* Atriplex hortensis* was observed in salt stress [17], while AsDREB from* Atriplex halimus* was induced by only dehydration [18]. PpDBF1 from* Physcomitrella patens* was induced under salt, dehydration as well as cold stress [19], while SbDREB2A from* Salicornia brachiata* was induced by NaCl, drought, and heat stress [20].

*Suaeda salsa* is a native halophyte in China for both industrial application and scientific research [21]. Fresh branches of* S. salsa* are highly valuable as a vegetable, and the seeds can produce edible oil [21]. It can grow both in saline soils and in the intertidal zone where soil salt reaches up to 3%. Treatment of* S. salsa* with 200 mM NaCl could significantly increase its growth and net photosynthetic rate [22]. The high salt tolerance might be partly the result of its efficient antioxidative system [22]. For instance, Mn-SOD and Fe-SOD activities in the leaves of* S. salsa* seedlings were significantly higher under NaCl stress conditions (100 mmol L-1) than those under non-NaCl stress conditions [22]. However, the mechanism of abiotic-stress-tolerance in* S. salsa* is still poorly understood. In the present study, we report the cloning and characterization of the* SsDREB* cDNA. Its expression pattern was investigated in response to exogenous ABA, salt, cold, and drought stress treatments. Overexpression of this cDNA in transgenic tobacco led to enhanced tolerance to salinity and dehydration stresses.

## 2. Materials and Methods

### 2.1. Plant Materials and Stress Treatment

Seeds of* S. salsa* were germinated and precultured in pots containing vermiculite with Hoagland nutrient solution in a growth chamber (20/25°C, 16 h light/8 h dark) under 250 mE · m^−2^ · s^−1^ light intensity.

Salinity, dehydration, and ABA stress treatments were performed on* S. salsa* by transferring 3-week-old seedlings in Hoagland nutrient solution supplemented with 250 mM NaCl, 20% PEG6000, and 100 *μ*m/L ABA, respectively. Low temperature treatments were performed by transferring plants to a growth chamber set to 4°C under the light and the photoperiodic conditions described above. Samples were harvested at 0, 0.5, 2, 4, 8, 12, and 24 h after treatment and immediately stored at −80°C for further study. All experiments were repeated in biological triplicates.

### 2.2. Gene Isolation and Sequencing Analyses

Total RNA was extracted from the leaves of* S. salsa*, treated with 400 mM NaCl for 6 h utilizing SV Total RNA Extraction Kit (Promega, USA) according to the instruction. The conserved AP2/ERF domain of DREB genes in* S. salsa* was amplified by primers DREB-C1 and DREB-C2, designed from the known DREB/CBF genes in the GenBank database. Isolation of the cDNA sequences was carried out using the RNA ligase-mediated rapid amplification of 5′ and 3′ ends (RLM-RACE) method, according to the GeneRacer Kit (Invitrogen, USA). Gene-specific nested primes 5GSP1, 5GSP2, 3GSP1, and 3GSP2 were designed based on the known genomic sequences. Sequences of all relevant primers are listed in Table 1.

The 5′- and 3′-RACE fragments were cloned into separate pGEM-T Easy plasmid vectors (Promega, USA) and sequenced. The cDNA sequences of* SsDREB* were amplified by PCR using the forward primer* SsDREB-G1* and the reverse primer* SsDREB-G2* (Table 1). PCR was performed with a 5-min 94°C denaturation step, followed by 30 cycles of 45 s at 94°C, 45 s annealing at 55°C, a 1-min extension at 72°C, and a final extension period of 10 min.

Sequence analyses were performed using the program BLASTX (National Centre for Biotechnology Information, USA). The ORF of* SsDREB* genes and the properties of protein encoded by them were predicted by DNAStar software. The conserved AP2 domains (Accession number: smart00380) were originally applied as a seed sequence to search the NCBI database (http://www.ncbi.nlm.nih.gov/) and 33 proteins were retrieved with an expected value of 100. Multiple alignments were prepared using ClustalW [23] using default parameters (gap opening penalty = 10, gap extension penalty = 0.2). The resulting alignments of complete protein sequences were used in MEGA (version 5) [24] for the construction of unrooted phylogenetic trees using the neighbor-joining (NJ) method according to Jones-Taylor-Thornton model with uniform rates among sites and complete deletion of gaps data. The reliability of the obtained trees was tested using bootstrapping with 500 replicates.

### 2.3. DRE Binding and Transcriptional Activity of FeDREB1 in Yeast

The DNA-binding activity of SsDREB protein was measured using a yeast one-hybrid system. Three tandem repeats of the core sequence of the DRE (TACCGACAT) and its mutant (mDRE) sequence (TATTTTCAT) were cloned into the Sac I/Spe I restriction sites of the plasmid pHIS2.1 cloning reporter vector upstream to the HIS3 minimal promoter according to the protocol described by Clontech (Clontech, Mountain View, CA, USA). The entire coding region of* SsDREB* was cloned into the Sma I site of the YepGAP expression vector containing no GAL4 activation domain (AD) [25]. The recombinant YepGAP expression vector containing* SsDREB* cDNA and the pHIS2.1 vector containing three tandem repeats of the DRE or mDRE were cotransformed into the yeast strain Y187. The growth status of the transformed yeast was compared on SD/-Leu-Ura-His+ 10 mM 3-AT plates to test the expression of the HIS reporter gene. Empty YepGAP was used as a negative control.

### 2.4. Gene Expression Assay by Quantitative Real-Time RT-PCR

Total RNA was extracted from the roots, stem, and leaves using SV Total RNA Extraction Kit (Promega, USA) according to the instruction. First-strand cDNA was produced from 1 *μ*g of RNA using PrimeScript RT reagent Kit (Takara, Dalian, China), according to the manufacturer's protocol. Each sample was amplified in biological and technical triplicate by quantitative real-time RT-PCR using a Roche 2.0 Real-Time PCR Detection System with the SYBR Green Supermix (Takara, Dalian, China). The reaction mixture was cycled as follows: 30 s denaturation at 95°C, then 40 cycles of 5 s at 95°C, 10 s at 60°C, and 20 s at 72°C. The amplification of* S. salsa Actin* gene (FJ587488) was used as the normalization control. The mRNA fold difference was relative to that of untreated samples used as calibrator. The relative quantification value for* SsDREB* was calculated by the 2^−ΔΔCT^ method [26]. All relevant primers used in this work are listed in Table 2.

### 2.5. Generation of Transgenic Tobacco

To generate transgenic plants,* SsDREB* cDNA was amplified using a specific primer pair: forward, 5′-GCCTCTAGAATGGCAGCTACAACAAT GGATATG-3′ (XbaI site underlined) and reverse, 5′-GCCCCCGGGTTAAGATGATGATGAT AAGATAGC-3′ (SmaI site underlined). The PCR product was fused into the binary plant transformation vector pCAMBIA2301 under the control of the CaMV 35S promoter. The constructs were mobilized to* Agrobacterium tumefaciens* strain EHA105. This* Agrobacterium* strain was used for transformation in tobacco leaf discs following the standard protocol [27]. The putative transgenic lines selected on medium containing hygromycin were confirmed by PCR with gene-specific primers.

The seeding of transgenic tobacco plants was selected on solid 1/2 MS medium containing 100 *μ*g/mL kanamycin under long-day condition (16 h light/8 h dark) at 25°C. The transgenic lines of tobacco plants were confirmed by qRT-PCR analysis.

### 2.6. Salinity and Drought Stress Tolerance Evaluation in Transgenic Plants

Independent homozygous transgenic plants lines and homozygous wild-type transgenic with pCAMBIA2301 empty vector (WT) were precultured in MS liquid medium for 4 days in growth chamber (20/25°C, 16 h light/8 h dark) under 250 mE/m2/s light intensity. Then, both plants were transferred in an aqueous MS medium supplemented with PEG6000 (0, 5, 10 15, and 20%) or NaCl (0, 50, 100, 150, 200, 250, and 300 mM) for 2 days. Leaves with and without stress treatments were sampled for physiological parameters.

### 2.7. Measurement of Photosynthetic and Chlorophyll Fluorescence Parameters

Leaf net photosynthetic rate (*P*_*n*_) was measured using a portable infrared gas analyzer (LI 6400XT portable photosynthetic system, Lincoln, USA). Chlorophyll index was measured using chlorophyll content meter (FMS-2 Pulse Modulated Fluorometer, Hansatech Inc., UK).

### 2.8. Measurement of Free Proline and Soluble Sugars Content

Fresh leaf material (0.3 g) was extracted with 5 mL of deionized water at 100°C for 10 min, and shaken with 0.03 g of permutit for 5 min. The extract was separated by centrifugation at 3,000 rpm for 10 min, and then the proline content of the aqueous extract was determined using the acid ninhydrin method. The organic phase was determined at 515 nm. The resulting values were compared with a standard curve constructed using known amounts of proline (Sigma).

Fresh leaf material (0.2 g) was extracted with 80% (v/v) ethanol at 70°C for 30 min. The extract was separated by centrifugation at 12,000 rpm for 10 min and diluted with water to 10 mL. Then, the soluble sugar content of the aqueous extract was determined using sulfuric acid anthrone colorimetric method. The resulting values were compared with a standard curve constructed using known amounts of sugar.

### 2.9. Semiquantitative RT-PCR for Expression Analysis of Downstream Genes of* SsDREB*

Semiquantitative RT-PCR amplification was performed with selected gene primers (Table 3), using the first strand cDNA, synthesized from RNA samples collected from WT and transgenic tobacco seedlings. The reaction mixture was cycled as follows: 3 m denaturation at 95°C, then 35 cycles of 45 s at 94°C, 45 s at 55°C, and 1 m at 72°C. The amplification of* S. salsa α*-tublin gene was used as the normalization control. PCR-amplified products were visualized on ethidium bromide-stained 1.5% agarose gels.

## 3. Results

### 3.1. Isolation and Phylogenetic Analysis of* SsDREB* cDNA

**Figure 1:**
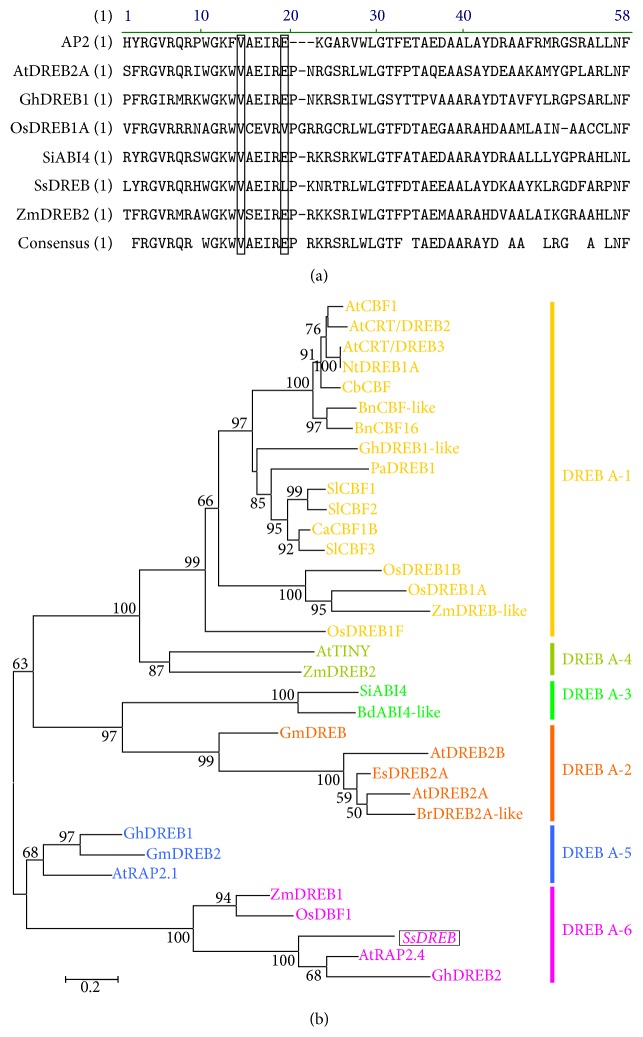
Conserved domain and phylogenetic analysis of SsDREB protein. (a) SsDREB protein has the same AP2 domain compared with other DREB proteins from* Arabidopsis thaliana*,* Zea mays*,* Gossypium hirsutum*,* Setaria italica*, and* Oryza sativa*. The 14th valine and the 19th leucine acid inside the AP2/ERF domain are presented in boxes. (b) Phylogenetic analysis of proteins from DREB subfamily. The number was the bootstrap value of the clade and low bootstrap values (<50) were removed from the tree. The accession number of each appended protein is as follows: AtCBF1 (AAC49662), AtCRT/DREB2 (AAD15976), AtCRT/DREB3 (AAD15977), NtDREB1A (ABD65969), CbCBF (AAR26658), BnCBF-like (AAL38242), BnCBF16 (AAM18960), GhDREB1-like (ABD65473), SlCBF3 (AAS77819), SlCBF2 (AAS77821), PaDREB1 (BAD27123), CaCBF1B (AAQ88400), OsDREB 1B (AAN02488), OsDREB 1A (AAN02486), ZmDREB2 (AAM80485), ZmDREB-like (AAN76804), AtRAP2.1 (AAC49767), AtRAP2.4 (AAC49770), SlCBF1 (AAK57551), AtTINY (CAA64359), AtDREB2B (BAA33795), AtDREB2A (BAA36705), ZmDREB1 (AAM80486), AtAP2 (AAC39489), ZmERF/AP2 (BAE96012), GmDREB (AAP83131), EsDREB2A (AAS58438), OsDBF1 (AAP56252), GhDREB1 (AAO43165.1), CbCBF25 (AAR35030), GmDREBa (AAT12423), BdABI4-like (XP_003568646), SiABI4-like (XP_004963859), BrDREB2A-like (XP_009125600), BpDREB (ABB89755.1), GmDREB (ABB36645), OsDREB1F (AAX23723), and GhDREB2 (AAT39542).

A full length-cDNA sequence, designated as* SsDREB*, was isolated from* S. salsa*. This cDNA is 1095-bp long corresponding to a protein of 364 amino acids.* SsDREB* possesses two regions rich in serine, one region rich in glutamine, and an acidic C-terminal sequence, PSXEIDW, which is known to function in transcriptional activation activity [25, 28]. The putative amino acid sequence showed that the* SsDREB* had a conserved EREBP/AP2 domain of 64 amino acids with valine (V) and leucine (L) at the 14th and 19th residues, respectively (Figure 1(a)). Phylogenic tree analysis of DREB proteins showed that* SsDREB*, together with* Arabidopsis *RAP2.4, ZmDREB1, OsDBF1, and ChDREB2, is attributable to the DREB (A-6) lineage (Figure 1(b)).

### 3.2. *SsDREB* Protein Specifically Binds to the DRE Element

**Figure 2:**
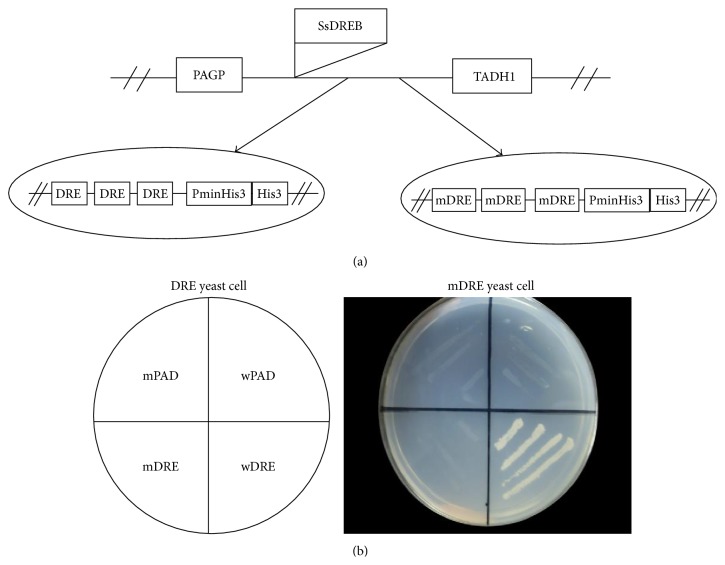
Analysis of SsDREB binding to the DRE element in the yeast one-hybrid system. (a) Construction of the YepGAP-*SsDREB* plasmid. The entire* SsDREB* coding region was fused to the activation domain of GAL4. Recombinant YepGAP-*SsDREB* or plasmid was transformed into yeast cells that are harboring two reporter genes under the control of either wild-type or mutant DRE motifs. PGAP and TADH1 indicate the promoter and terminator of ADH1 gene, respectively. (b) Transformed yeast cells were examined for growth on selective medium (SD−His+10 mM 3-AT) at 30°C (left). Left panel shows the position of each transformed yeast cell. The empty YepGAP (PAD) was used as a control. wDRE and wPAD indicate yeast cells harboring DREB proteins and DRE-controlled reporter genes, while mDRE and mPAD indicate yeast cells harboring DREB proteins and mDRE-controlled reporter genes.

To verify the possible binding function between SsDREB protein and DRE element, the recombinant plasmid pAD-SsDREB was separately transformed into yeast strain Y187 containing the reporter genes HIS3 under the control of DRE. As negative controls, pAD-SsDREB was also separately transformed into Y187 harboring the reporter genes HIS3 under the control of a mutant DRE (mDRE) (Figure 2(a)). These results suggested that the DRE::pAD-SsDREB transgenic yeast cells grew well on SD/-His 10 mM 3-AT, whereas the yeast cells harboring mDRE::pAD-SsDREB transgenic yeast cells could not grow on the same medium (Figure 2(b)). These results strongly indicated that the SsDREB can bind the normal DRE element exclusively to drive target gene expression* in vivo*.

### 3.3. Expression of* SsDREB* in Response to Various Abiotic Stresses

**Figure 3:**
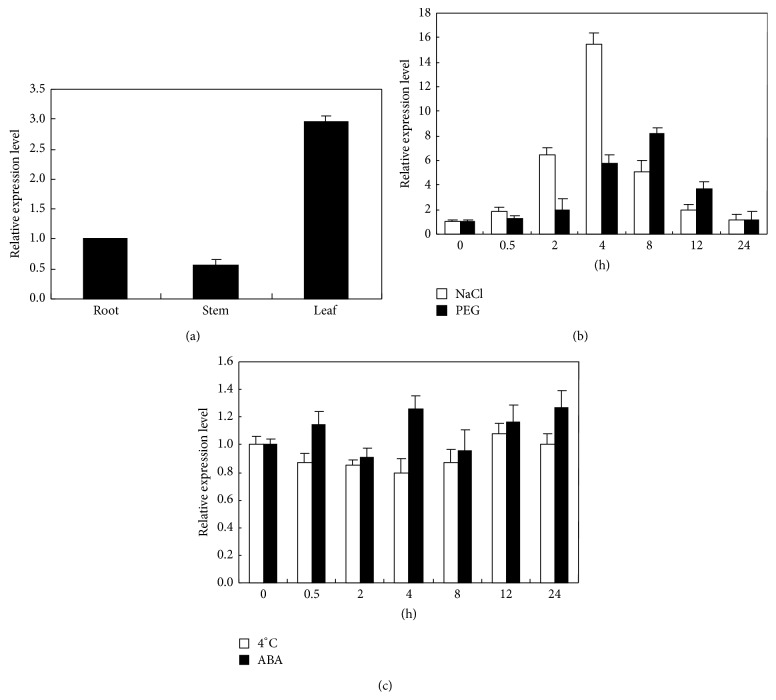
Quantitative real-time PT-PCR analysis of* SsDREB*. (a) Transcript levels of* SsDREB* in the roots, stems, and leaves of untreated plants. (b), (c) The relative expression level of* SsDREB* in* S. salsa* leaves at indicated time points exposed to salinity stress (250 mM NaCl), dehydration stress (20% PEG), low temperature (4°C), and 100 *μ*M ABA, respectively. Columns indicate relative expression levels of* SsDREB* normalized against levels of SsActin as calculated by real-time qRT-PCR (mean ± SE of three biological replicates).

The expression pattern of* SsDREB* in different organs of* S. salsa* was examined under normal conditions. The expression level of MsDREB2C was highest in leaves followed by roots and stem (Figure 3(a)). Therefore, expression of* SsDREB* in leaf was investigated under different abiotic stresses. Quantitative reverse transcription-PCR (qRT-PCR) revealed that the transcript of* SsDREB* was induced by salt and drought stress.* SsDREB* expression was induced by salt treatment at 0.5 hours after treatment and peaked at 4 h, with the highest abundance of about 16-fold increase (Figure 3(b)). The expression increased slowly from 0.5 h but rapidly peaked at 8 h and then decreased gradually under mimic dehydration stress (Figure 3(b)). Under cold stress (4°C) treatment,* SsDREB* expression was gradually declined and then slightly recovered after 4 h after treatment (Figure 3(c)). Similarly, there was no significant expression change of* SsDREB* after exogenous ABA application, indicating that StDREB1 may function in an ABA-independent signaling pathway (Figure 3(c)).

### 3.4. Confirmation of Putative Transgenic Tobacco Plants Expressing* SsDREB*

**Figure 4:**
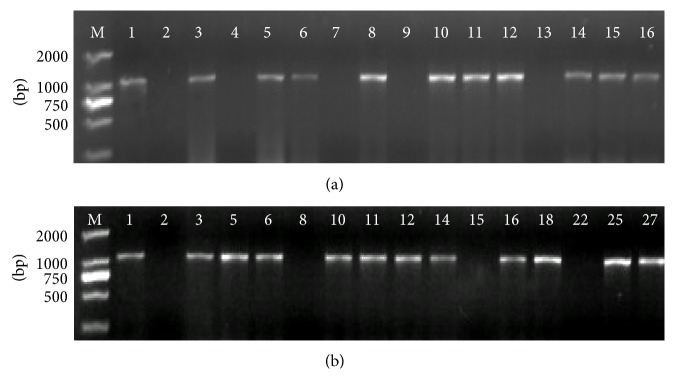
(a) PCR amplification of the specific* SsDREB* gene from genomic DNA of the transgenic lines. (b) RT-PCR analysis of* SsDREB* expression in transgenic lines. (*M* marker DL2000, 1 CK+, 2 WT, 3–27 transgenic lines).

The putative transgenic lines selected on medium containing hygromycin were confirmed by PCR with gene-specific primers using primers the pCAMBIA2301 binary vector corresponding to sequences flanking the* SsDREB* cDNA. As expected, a PCR product of 1095 bp was obtained (Figure 4(a)). PCR-positive plants were successfully transferred to green house for further analysis. Positive transgenic lines also showed expression of* SsDREB* by semiquantitative RT-PCR, whereas expression of* SbDREB* was not observed in WT plants (Figure 4(b)). No phenotypic modification such as dwarfism was noticed in these* SsDREB* transgenic plant lines.

### 3.5. Tobacco Plants Overexpressing* SsDREB* Enhance Salinity and Dehydration Tolerance

#### 3.5.1. Morphological Features of Plants

**Figure 5:**
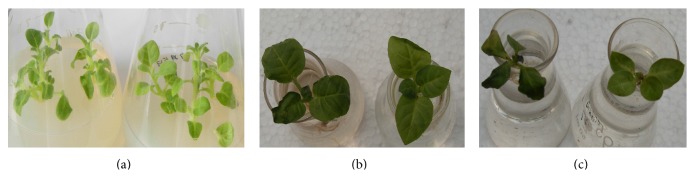
Phenotype of transgenic tobacco plants under normal and stress condition. (a) Under normal condition. (b) Treated with 300 mM for 2 days. (c) Treated with 20% PEG for 2 days.* Left*: WT plant;* right*: transgenic plan.

All of the transgenic lines and WT tobacco plants grew well under normal condition (Figure 5(a)). After dehydration and salinity treatment, decrease in leaf size was observed in both transgenic and WT plants. The salt stress proved more detrimental in the WT plants as compared to transgenic seedlings. At 300 mM NaCl, the transgenic plants showed better growth under salt stress with larger leaf area and higher turgor maintenance pressure (Figure 5(b)) as compared to WT. At 20% PEG, the transgenic plants showed significantly better growth under stress with larger leaf area and higher turgor maintenance pressure (Figure 5(c)) as compared to WT.

#### 3.5.2. Photosynthesis and Chlorophyll Fluorescence Parameters

**Figure 6:**
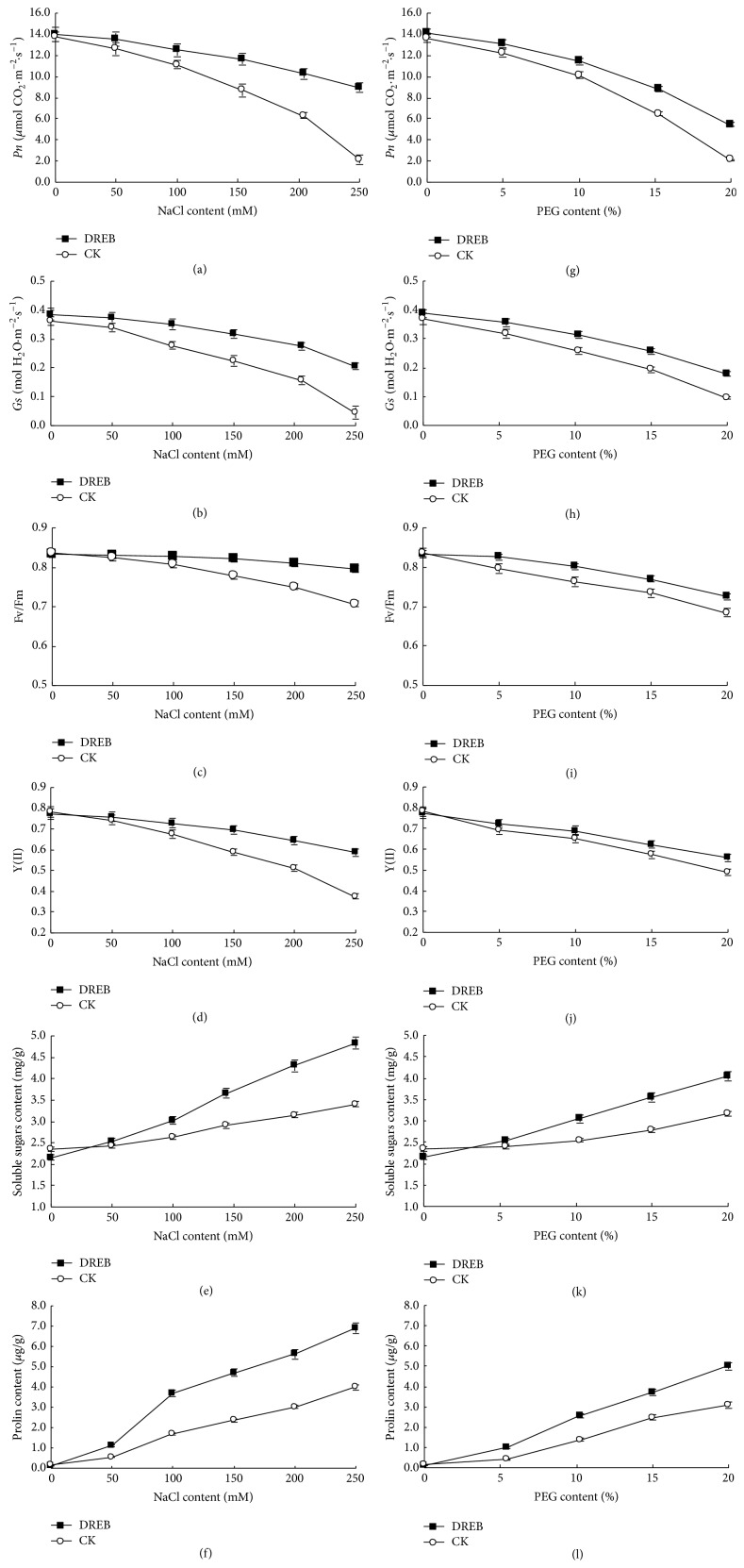
Salinity and dehydration tolerance of transgenic tobacco. (a)–(f) Salinity tolerance of transgenic plants. (g)–(l) Dehydration tolerance of transgenic plants. (a), (g) Net photosynthesis rate (*P*_*n*_). (b), (h) Stomatal conductance (*G*_*s*_). (c), (i) Maximal PS II quantum (Fv/Fm). (d), (j) Effective PS II quantum yield (Y II). (e), (k) Soluble sugars content. (f), (l) Proline content. For (a)–(l), each data point is means from three replicates ± SE. Bars indicate SE.

Net photosynthesis rate (*P*_*n*_) and stomatal conductance (*G*_*s*_) in WT and transgenic plants were similar under control condition. Net photosynthesis rate (*P*_*n*_) was 13.8 and 14.0 *μ*mol CO_2_ · m^−2^ · s^−1^ in WT and transgenic plants while stomatal conductance was 0.37 and 0.38 *μ*mol CO_2_ · m^−2^ · s^−1^ under control condition. Under salinity stress net photosynthesis rate and stomatal conductance reduced drastically as compared to control conditions in WT and transgenic lines. Transgenics showed significantly higher net photosynthetic rate and stomatal conductance at all five NaCl concentration gradients, compared to WT plants indicating. Net photosynthesis rate was reduced to 12.6, 11.1, 8.7, 6.3, and 2.2 *μ*mol CO_2_ · m^−2^ · s^−1^ in WT plants 2 days after 50, 100, 150, 200, and 250 mM NaCl treatment, respectively. But transgenic plants maintained net photosynthesis rate at 13.6, 12.5, 11.6, 10.3, and 8.9 *μ*mol CO_2_ · m^−2^ · s^−1^ in the same treatment (Figure 6(a)). In addition, stomatal conductance was reduced to 0.34 and 0.04 mol H_2_O · m^−2^ · s^−1^ in WT plants, while transgenic plants maintained around 0.37 and 0.21 H_2_O · m^−2^ · s^−1^ days after 50 and 250 mM NaCl treatment, respectively (Figure 6(b)). Similarly, transgenics showed significantly higher net photosynthetic rate and stomatal conductance at all four PEG concentration gradients, compared to WT plants (Figures 6(g)-6(h)). The results showed that* SsDREB* transgenic plants showed higher tolerance to salt stress.

Chlorophyll fluorescence parameters were also investigated. Maximal PS II quantum efficiency (Fv/Fm) and effective PS II quantum yield (Y II) in WT and transgenic plants were similar under control condition. Fv/Fm was 0.84 and 0.83 in WT and transgenic plants while Y (II) was 0.78 and 0.77 under control condition. The transgenics showed higher Fv/Fm and Y (II) at all five NaCl concentration gradients, compared to WT plants (Figures 6(c)-6(d)). Fv/Fm was reduced to 0.82, 0.81, 0.78, 0.75, and 0.71 in WT plants 2 days after 50, 100, 150, 200, and 250 mM NaCl treatment, respectively. But transgenic plants maintained Fv/Fm at 0.83 under 50 and 100 mM NaCl stress and then drop slightly to 0.82, 0.81, and 0.80 under 150, 200, and 250 mM NaCl treatment (Figure 6(c)). In addition, Y (II) was reduced to 0.74 and 0.38 in WT plants, while transgenic plants were maintained around 0.76 and 0.59 2 days after 50 and 250 mM NaCl treatment, respectively (Figure 6(d)). Similarly, the transgenics showed higher chlorophyll fluorescence parameters at all four PEG gradients, compared to WT plants (Figures 6(i)-6(j)), indicating that expression of* SsDREB* in transgenic tobacco enhanced abiotic tolerance.

#### 3.5.3. Proline and Soluble Sugar Content

Proline and soluble sugar content accumulate in plants subjected to salinity and dehydration stress conditions to confer stress tolerance in both transgenic and WT plants. The contents of soluble sugar and free proline in transgenic plants were slightly richer than that of the WT plants with all salinity and dehydration stress, demonstrating that the overexpression of* SsDREB* gene could enhance plant salinity and dehydration tolerance in transgenic tobacco (Figures 6(e)-6(f), 6(k)-6(l)).

### 3.6. Overexpression of StDREB1 Activates the Expression of Stress-Responsive Genes

**Figure 7:**
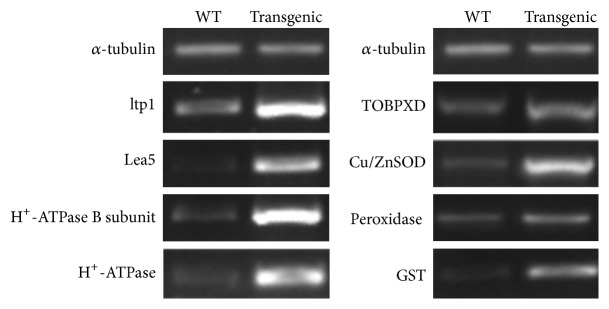
Semiquantitative RT-PCR of stress-responsive genes.

Given that the* SsDREB* transgenic plants showed enhanced tolerance to salinity and drought and freezing stress, we decided to quantify the molecular responses of eight stress-responsive genes in the transgenic lines to see the level of expression under stress conditions. Semiquantitative RT-PCR analyses of these target genes were performed for the WT and for the* SsDREB* transgenic tobacco plants. An increase in transcription level of these genes was noticed in almost all transgenic plants cultivated under standard growth conditions in comparison to those in WT ones (Figure 7). This most significant increase was in expression of* ltp1*,* Lea5*, and* H* ^*+*^ *-ATPase *genes, while expression of* Cu/Zn SOD*,* TOBPXD*, and* GST* was slightly higher in transgenic plants under the same situation. All these findings strongly suggested that* SsDREB* might upregulate the expression of stress-related functional genes.

## 4. Discussion

This study describes the isolation and characterization of a DREB factor from halophyte* Suaeda salsa*, termed* SsDREB*. To date, only few efforts are made in halophytes in response to salt stress. The AhDREB1 from* Atriplex hortensis* expression was observed in salt stress [17], while AsDREB from* Atriplex halimus* was induced by dehydration but not in salt stress [18]. PpDBF1 from* Physcomitrella patens* was induced under salt, dehydration as well as cold stress [19]. DREB2-type TFs SbDREB2A from halophytic plants* Salicornia brachiata* was induced by NaCl, drought and heat stress [20].

The sequence analysis of* SsDREB* identified an AP2/ERF domain of 64 amino acids that is predicted to fold into a structure containing three anti-parallel *β*-sheets and one *α*-helix.* SsDREB* possessed two regions rich of serine, one region rich of glutamine, and an acidic C-terminal sequence, PSXEIDW, which is known to function in transcriptional activation activity [25, 28]. This structure is thought to play a key role in recognizing and binding to specific cis-elements [7]. Sequence alignment and phylogenetic analyses revealed that the* SsDREB* grouped with the DREB (A-6) lineage. In this study, the DSAW and LWSY motif, the conserved sequences in A1-subgroup (DREB1) [29], was not found in* SsDREB*.

A number of reports have suggested that Val14 and Glu19 in the AP2/ERF domain are essential for specific binding to DRE [7, 25]. The absolutely conserved 14th valine residue, an important site that acts in DNA binding, has also been found in the AP2/ERF domain of SsDREB protein [7]. However, the 19th glutamic acid residue is replaced by leucine residue in the* SsDREB* (Figure 1(a)). Similar amino acid changes have also been observed in other plant species. In rice, wheat, and barley, the DREB1-type factors harbor a valine residue at position 19 in the AP2/ERF domain [30, 31]. The Glu (E) 19 is also replaced by Gln (Q) in potato (*Solanum tuberosum* L.) and by His (H) in Buckwheat (*Fagopyrum esculentum*). In* Broussonetia papyrifera*, the 19th glutamic acid residue in BpDREB2 protein was replaced by leucine residue, and the DNA binding assay in the yeast one-hybrid system suggested that the 14th residue is more crucial than the 19th residue in the DRE binding activity of DREB [32]. Other research also reported that mutation in the 19th residue had little effect on DRE binding activity [33]. Our DNA binding assay in the yeast one-hybrid system also suggested that the change in the 19th residue had little effect on DRE binding activity (Figure 3(b)). The mutation of the 19th residue in the AP2/ERF domain indicated that the conserved 14th valine residue may be crucial in the regulation of the DRE binding activity of DREB.

It was reported that the expression of DREB1 (A-1) genes was induced by low temperature, whereas the expression of DREB2 (A-2) genes was attributed to dehydration or salt stress [25]. Quantitative real-time RT-PCR analysis showed that the transcripts of the* SsDREB* were induced by drought and salt stress but not by cold treatment, which is in agreement with previous reports describing the role of DREB factors in plant response to abiotic stress [4, 34]. However, the transcripts of the* SsDREB* were not induced by exogenous ABA application in* S. salsa* (Figure 5). Many studies showed that ABA phytohormone, whether endogenous or exogenous, is involved in several physiologic processes and in the adaptation of plants to different abiotic stresses and plays a crucial role in inducing the expression of some stress-responsive genes [35, 36]. Transcript accumulation of StDREB1 gene from potato (*Solanum tuberosum* L.) was significantly induced by exogenous application of 50 *μ*M ABA, indicating that StDREB1 may function in an ABA-dependent signaling pathway [19]. The transcripts of the FeDREB1 from buckwheat were induced by low-/high-temperature treatment, drought stress, and exogenous ABA application [14]. However, several exceptions regarding this expression pattern have been reported. For instance,* Glycine max* GmDREB2A, a member of the DREB (A-2) group was highly induced not only by dehydration and heat but also by low temperature [37], and PeDREB2 from* Populus euphratica *was induced by drought and salt, as well as cold stress [38]. Moreover, a ZmDBP4, belonging to DREB (A-1) gene, was activated by cold and drought, but not ABA [39]. GmDREB2 and BpDREB2 were also reported not to be responsive to ABA treatment [32, 40]. Our research results indicated that* SsDREB* genes were not responsive to ABA treatment, which suggests that* SsDREB* genes are involved in the dehydration and salinity stress responses through ABA-independent pathways.

Morphological and physiological parameters are actual indicators of stress endurance of transgenic plants. The* SsDREB* transgenic plants imparted both salinity and dehydration tolerance with better morphological growth like larger leaf area and higher turgor maintenance pressure. In contrast to the data reported by Yamaguchi-Shinozaki and Shinozaki [41], the overexpression of StDREB1 gene in transgenic plants did not show any phenotypic changes such as dwarfism. Fluorescence-based photosynthetic activity of leaves plays an important role in adaptation to abiotic stress. Under salinity and dehydration stress, the* SsDREB* transgenic plants kept higher photosynthesis and chlorophyll fluorescence parameters than WT plants, revealing better abiotic stress tolerance.

During stress conditions, proline helps the plant cell by stabilizing subcellular structures such as membranes and proteins, scavenging free radicals and buffering cellular redox potential [42]. Previous studies reported that AtDREB1 could enhance the drought tolerance of transgenic Arabidopsis by activating the expression of downstream genes involved in sugar biosynthesis and proline biosynthesis [43]. Transgenic tobacco overexpressing* SsDREB* accumulated higher free proline and soluble sugar than WT plants under salinity and dehydration stress, revealing the improved salinity and drought tolerance of the transgenic plants. Similarly, overexpression of SbDREB in* Salicornia brachiata* [20] and OsDREB2A in rice [44] also resulted in higher accumulation of proline under salt stress.

The constitutive expression of* SsDREB* conferred improved tolerance to drought and salinity in transgenic plants, possibly because of the overexpression of stress-inducible DREB2-responsive genes. LEA proteins were quite hydrophilic and were believed to protect plant cells from these stresses. Furthermore, the activity of LEA genes was associated with cold stress in plants [45]. In this study, expression level of LEA5 increased significantly in transgenic plants, indicating that SsDREB had activated the expression of downstream genes like LEA5. The expression of glutathioneS-transferase (GST) and superoxide dismutase (SOD) was not high in transgenics, indicating that* SsDREB* was not responsive to oxidative stress.

In conclusion, a novel* SsDREB* transcription factor was cloned from* Suaeda salsa *andclassified in the A-6 group based on phylogenetic characterization. Yeast one-hybrid assays verified that SsDREB protein specifically binds to the DRE element. Real-time RT-PCR showed that* SsDREB* was significantly induced under salinity and drought stress. Overexpression of* SsDREB* cDNA in transgenic tobacco plants exhibited an improved salt and drought stress tolerance, suggesting that the* SsDREB* transcription factor is involved in the regulation of abiotic stress tolerance in tobacco by the activation of different downstream gene expression.

## Figures and Tables

**Table 1:** Primers used for RACE-PCR amplification.

Primer name	Oligonucleotides (5′-3′)	Use
DREB-C1	TGGGG KAAR TGGGTYGCHGARAT YCG	AP2/ERF domain
DREB-C2	ACDGADGARTGNAGWGGYT TRTA	AP2/ERF domain
5′AAP:	GGCCACGCGTCGACTAGTACGGGIIGGGIIGGGIIG	5′ Universal Primer
5′AUAP	GGCCACGCGTCGACTAGTAC	5′ Universal Primer
5GSP1	TGACCAAACTAGACTCCCTCTAACA	*SsDREB*a 5′ RACE
5GSP2	AGTATTG CTCCGCTCCTAACTCTT	*SsDREB*a 5′ RACE
3′AUAP	GGCCACGCGTCGACTAGTAC	3′ Universal Primer
3GSP1	GA CTACCCAAGAACCGAACCCGGTT	*SsDREB*a 3′ RACE
3GSP2	GGTTATGGCTTGGATCCTTCGATA C	*SsDREB*a 3′ RACE
*SsDREB*-G1	ATGGCAGCTACAACAA TGGATATG	cDNA
*SsDREB*-G1	TTAAGATGATGATGATAAGATAGC	cDNA

**Table 2:** Primers used for qRT-PCR amplification.

Primer Name	Oligonucleotides (5′-3′)	Use
*SsDREB*-R1	AGAGGGAGTCTAG TTTGGTCATT	Real-time qRT-PCR
*SsDREB*-R2	TTTGGAGCCCCTACAATTTC	Real-time qRT-PCR
SsACTIN-R1	ACCGTTCCAATCTATGAGG	Reference gene
SsACTIN-R2	CGTAAGCCAACTTCTCCT	Reference gene

**Table 3:** Primers of downstream genes of *SsDREB* for semiquantitative RT-PCR.

Gene (GeneBankID)	Oligonucleotides (5′-3′)
*α*-tublin (AJ421412)	TAACCATCATAGAAGAGGGTTC
	GCAATCCTTCTTGACAATGAGG

Glutathione S-transferase (D10524)	TTGGCCTTCTACTTCCATCC
	TGTCAACTGCAACCATGAGAG

Cu/ZnSOD (EU123521)	TGTCACGGGACCACATTAC
	CACCAGCATTTCCAGTAGC

Lea5 (AF053076)	GTGCCAGGTGGAGTGAGAGG
	GGGACGTGGTATGGTAACCA

lipid transferase (ltp1) (X62395)	AATAGCTGGGAAAATTGCATG
	CAGTGGAAGGGCTGATCTTG

H^+^-ATPase B subunit (AF220611)	TCTTCACCAGTCCAGCCTGAC
	GAAGGAACATCTGGAATTGAC

H^+^-ATPase (X66737)	TCAGCAGGAATGATGTCTCC
	TCATGGAAGCTGCTGCTGTC

Peroxidase (AY032675)	AGGGGAAATGTTATTGTCTCC
	CACATTGGGAAGTACCACTAG

TOBPXD (D11396)	GAAATCCTGGCTCCGCTCTG
	TGGAGTTGCCTTGGTAAGAG
